# Preparation of biodegradable poly(lactic acid)-*b*-polyamide 4 block poly(ester amide) and its electrospun fibers

**DOI:** 10.1039/d6ra01092j

**Published:** 2026-04-20

**Authors:** Fan Mo, Chen Tian, Rongrong Ji, Shixing Dong, Linwei Liang, Ling Zhou, Hao Wu, Xipo Zhao

**Affiliations:** a Hubei Provincial Key Laboratory of Green Materials for Light Industry, Hubei University of Technology Wuhan Hubei 430068 P. R. China xpzhao123@163.com; b New Materials and Green Manufacturing Talent Introduction and Innovation Demonstration Base, Hubei University of Technology Wuhan Hubei 430068 P. R. China; c Hubei Longzhong Laboratory Xiangyang Hubei 441000 P. R. China

## Abstract

Poly(ester amide) (PEA) contains both ester bonds (–COO–) and amide bonds (–NHCO–) within its molecular backbone. Through strategic monomer selection and molecular structure design, PEAs have the potential to simultaneously exhibit excellent biodegradability and mechanical properties, making them a promising class of materials with significant research interest and application potential. In this study, poly(lactic acid) (PLA) was synthesized from l-lactic acid *via* melt polycondensation, followed by terminal thiolation modification. Subsequently, a biodegradable PLA-*b*-PA4 block poly(ester amide) was prepared through a thiol–ene click reaction with the synthesized polyamide 4 (PA4). The molar ratios of polyester to polyamide repeating units in the copolymers were 11 : 26 and 23 : 26. Comprehensive structural characterization of thiol-terminated PLA and the synthesized block poly(ester amide) was conducted using MALDI-TOF MS, FTIR, Raman, XPS, and ^1^H-NMR spectroscopy. Systematic study revealed that variations in PLA chain length significantly influenced the properties of the PEA, with the PLA and PA4 segments undergoing phase separation in the melt and exhibiting mutually suppressed crystallization behavior. Electrospinning was employed to process the PEA. The electrospinning process reduces the crystallinity of both PLA and PA4. The morphology and properties of the electrospun fibers were influenced by the PLA block ratio. Poly(ester amide)s containing short-chain PLA segments produced electrospun fibers with larger diameters, exhibiting better mechanical strength and ductility. As the PLA chain length increased in the copolymer, bead formation appeared in the electrospun fibers, accompanied by a decrease in fiber diameter and a deterioration in mechanical performance. This study provides experimental evidence and theoretical insights into the structure–property relationships of fully biodegradable block poly(ester amide)s and their application in electrospinning.

## Abstract 2

Biodegradable PLA-*b*-PA4 copolymers were synthesized *via* thiol-ene click chemistry. The synergistic effect of block components facilitates the fabrication of stable electrospun fibers with tunable morphology and mechanical properties.

## Graphical abstract

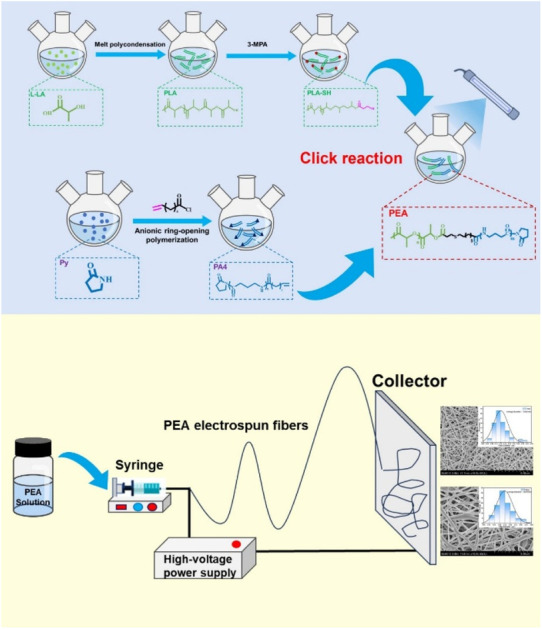

## 1. Introduction

In recent decades, biodegradable polymers have attracted widespread attention from both academia and industry as sustainable alternatives to petroleum-based polymers. Replacing conventional commercial polymers with biodegradable ones has become a crucial strategy for addressing the global issue of plastic pollution.^1–4^ Poly(ester amide)s contain repeating ester bonds (–COO–) and amide bonds (–NHCO–) within their molecular chains. They combine the biodegradability and biocompatibility conferred by the aliphatic ester groups with the high thermal stability, high Young's modulus, and high strength imparted by the strong intermolecular interactions established through the hydrogen-bonding network between amide groups. The properties of PEAs can be tailored by varying the types of copolymerized monomers and adjusting the ester-to-amide ratio within the polymer backbone.^5–8^ Block-structured PEAs possess unique chemical architectures that often exhibit properties distinct from those of alternating and random PEAs. They retain the characteristics of each individual block and can serve as compatibilizers in polymer blends or as thermoplastic elastomers. Additionally, the ordered nanostructures self-assembled from block PEAs have been utilized to tailor optical properties for advanced applications.^9–12^

Due to the incompatibility between polymer segments, block copolymers can undergo nanoscale microphase separation during electrospinning, forming ordered structures with various morphologies such as lamellar, bead-like, and spherical domains. These structures are beneficial for applications in separation membranes, sensors, and tissue engineering. Moreover, the microphase-separated morphology can induce specific surface features on the fibers such as rough textures and nanopores, thereby increasing the specific surface area and enhancing performance in filtration and drug delivery. However, current electrospinnable block copolymers are predominantly petroleum-based and fail to meet the demands of sustainable applications. Electrospun membranes based on block PEAs hold promise by combining the biodegradability and tunable properties of PEAs. By adjusting the molecular structure of PEAs in the spinning solution, it is possible to precisely control and balance the strength and toughness of the resulting fibrous membranes.^13–16^

Polylactic acid is a biodegradable thermoplastic polyester known for its excellent compostability, biocompatibility, mechanical properties, and processability. It has been widely applied in various fields, including the food industry and biomedical applications.^17,18^ However, the relatively weak intermolecular interactions between PLA chains result in a slow crystallization rate and low melt strength, while its inherent brittleness limits its application in certain fields. Additionally, the simple molecular structure of PLA, with a limited number of reactive functional groups, restricts its potential for further chemical modification and functionalization.^19,20^ PA4 can be synthesized from the bio-based γ-aminobutyric acid derived from l-glutamic acid. It features closely packed molecular chains and strong hydrogen bonding interactions, which facilitate crystallization. PA4 exhibits good moisture absorption and excellent thermal stability, with Young's modulus and elongation at break comparable to those of Nylon 6.^21,22^ Previous studies have confirmed that polyamide 4 exhibits biodegradability in diverse environments such as soil, seawater, activated sludge, and *in vitro* degradation systems. PA4 is currently recognized as the only biodegradable polyamide, and poly(ester amide)s derived from PA4 have also been reported to retain biodegradability.^23–27^ However, the excessively dense hydrogen bonding results in a melting temperature close to its thermal decomposition temperature, leading to a narrow processing window and making thermal processing and molding challenging.^28,29^

PLA and PA4 exhibit complementary properties, and the preparation of block copolymers from these two biodegradable materials can integrate the advantages of PEAs, achieving synergistic performance improvement and a full carbon cycle. However, reports on copolymers based on PLA and PA4 remain limited, primarily due to the poor solubility of PA4 in common organic solvents such as toluene, chloroform, and tetrahydrofuran. Additionally, PA4 is typically synthesized *via* anionic ring-opening polymerization conducted at 30–50 °C, which differs significantly from the ring-opening polymerization conditions of lactide (160–180 °C), posing challenges for copolymerization.^30^ Kim *et al.*^31^ reported a copolymer of polylactic acid and polyamide 4 synthesized using butyllithium as a catalyst. In this process, the terminal acyl lactam groups of PA4 undergo deprotonation and subsequently replace the end groups of PLA. The reaction was carried out at −78 °C, involving relatively harsh conditions, and the use of butyllithium also poses safety concerns. Chen *et al.*^32^ employed 2,2′-dithiodiethanol to initiate the ring-opening polymerization of lactide, yielding a symmetrical polylactic acid containing disulfide bonds (PLA–SS–PLA). Subsequently, the disulfide bonds were reduced using the strong reducing agent tributylphosphine to obtain thiol-terminated PLA. This thiol-terminated PLA was then copolymerized with vinyl-terminated polyamide 4 *via* thiol–ene click chemistry. However, the synthesis of thiol-terminated PLA involves lactide and 2,2′-dithiodiethanol, which are relatively costly, and the use of tributylphosphine presents significant safety risks due to its strong irritant properties. In the synthesis of thiol-terminated polylactic acid, the melt polycondensation method demonstrates significant advantages over ring-opening polymerization. This approach offers higher cost-effectiveness and a simplified operational process, while yielding purer products. Moreover, the melt polycondensation reaction does not require toxic solvents or initiators, exhibiting clear benefits in terms of process safety and environmental friendliness.^33^ Current studies have yet to report on the preparation of thiol-terminated polylactic acid *via* melt polycondensation and its subsequent chemical copolymerization with polyamide 4. Additionally, there is a lack of systematic research on biodegradable block polyesteramides in the context of electrospinning applications.

In this study, lactic acid and 3-mercaptopropionic acid were used as monomers to synthesize thiol-terminated poly(lactic acid) *via* a melt polycondensation method. Subsequently, a novel poly(ester amide) material was obtained through copolymerization with poly(butyrolactam) *via* a thiol–ene click reaction. The chemical structures of the thiol-terminated poly(lactic acid) and the resulting copolymers were characterized by MALDI-TOF MS, FTIR, Raman spectroscopy, and ^1^H NMR. By adjusting the polyester-to-amide ratio at the molecular level, the effects of chemical composition on thermal and crystallization properties were investigated. The copolymers were further applied to electrospinning, where the effect of the polymer solution concentration on fiber morphology was investigated. In addition, the influence of PLA chain length on fiber morphology, crystallinity, and mechanical properties was systematically analyzed. This work fills the existing gap in understanding the relationship between molecular structure, processing conditions, and final properties of block poly(ester amide)s in electrospinning applications, and provides a new processing strategy and theoretical basis for the development of high-performance PLA-based materials and poly(ester amide)s.

## 2. Experimental

### 2.1 Materials

l-Lactic acid (l-LA, Jindan, ≥95%), 3-mercaptopropionic acid (3-MPA, Macklin, ≥98%), stannous octoate (Macklin, ≥95%), *p*-toluenesulfonic acid (*p*-TA, Macklin, ≥99%), dichloromethane (DCM, SCR, ≥99.5%), 2-pyrrolidone (Py, Aladdin, ≥99%), 10-undecenoyl chloride (Adamas, ≥99%), formic acid (SCR, ≥99.5%), methanol (SCR, ≥99.5%), potassium *tert*-butoxide (*t*-BuOK, Meryer, ≥99%), hexafluoroisopropanol (HFIP, Adamas, ≥99%), 2,2-dimethoxy-2-phenylacetophenone (DMPA, Aladdin, ≥99%), tetrahydrofuran (THF, SCR, ≥99.5%).

### 2.2 The synthesis of polylactic acid

Fifty grams of l-LA were weighed into a three-neck flask and purged with nitrogen for 3 minutes. A prepolymerization was carried out under reduced pressure at 120 °C for 2 hours. Subsequently, 0.25 g of Sn(Oct)_2_ catalyst (0.5 wt% relative to lactic acid) was added. The temperature was then raised to 180 °C, and polymerization was continued under reduced pressure for 1 hour and 3 hours, respectively. The resulting products were designated as PLAL and PLAH accordingly.

The crude PLAL was dissolved in dichloromethane (DCM) and then purified *via* dialysis against deionized water for 48 hours using a dialysis membrane. Subsequently, the solvent was removed under vacuum to yield the purified PLAL. The PLAH was purified using a precipitation method. Specifically, the PLAH was dissolved in DCM and then added dropwise into an excess of cold methanol to induce precipitation. The precipitate was collected by suction filtration and washed several times with a methanol/water mixture. Product was dried under vacuum at room temperature for 24 hours to obtain the purified PLAH.

### 2.3 Synthesis of thiol-terminated polylactic acid

Ten grams of PLA were weighed and heated to 160 °C under a nitrogen atmosphere until fully melted. Then, 1.88 g of 3-mercaptopropionic acid (3-MPA) was added at a molar ratio of 1.8 : 1 relative to hydroxyl groups, along with 0.12 g of *p*-toluenesulfonic acid (*p*-TA, 1 wt% relative to PLA). The reaction was carried out under reduced pressure at 160 °C for 2 hours. The product was dissolved in dichloromethane (DCM), precipitated with methanol, washed with water, and vacuum-dried for 24 hours. The thiol-terminated products of PLAL and PLAH were designated as PLAL-SH and PLAH-SH, respectively.

### 2.4 Synthesis of alkenylated PA4

Thirty grams of pyrrolidone and 3 grams of potassium *tert*-butoxide (*t*-BuOK, 10 wt% relative to Py) were reacted under reduced pressure at 90 °C for 1.5 hours. After purging with nitrogen for 3 minutes, 0.9 grams of 10-undecenoyl chloride (initiator, 3 wt% relative to Py) was added, and the reaction proceeded at 55 °C for 1 hour. The product was dissolved in formic acid, precipitated with methanol, and vacuum-dried for 24 hours to obtain vinyl-terminated PA4.

### 2.5 Synthesis of PEA copolymer

PLA-SH and twice the molar amount of PA4 relative to the thiol groups were dissolved in HFIP (20 wt% solution) and stirred at 50 °C until fully dissolved. The photoinitiator DMPA (molar ratio to PLA 1 : 1) was added, and the solution was purged with nitrogen for 10 min, then stirred at 50 °C until fully dissolved. Subsequently, the reaction mixture was irradiated with ultraviolet light (365 nm) for 3 h at 50 °C in a sealed and light-shielded chamber. The product was precipitated with methanol, unreacted caprolactam was removed by washing with a 10 wt% CaCl_2_/methanol solution, and unreacted PLA was removed by washing with dichloromethane. The final product was vacuum-dried for 24 h. The copolymers of PLAL/PLAH with PA4 were designated as PEAL and PEAH, respectively. The polyester-to-amide molar ratios (PLA-to-PA4) were 11 : 26 for PEAL and 23 : 26 for PEAH.

### 2.6 Preparation of PEA electrospun fiber membranes

The polymer was dissolved in HFIP to prepare the spinning solution with a concentration of 12 wt%. Before electrospinning, the solution was uniformly dispersed by ultrasonic agitation and then allowed to rest for degassing. The degassed solution was loaded into a 10 mL syringe equipped with a stainless steel needle (inner diameter 0.6 mm). Electrospinning was carried out at room temperature under a voltage of 25 kV. The syringe feed rate was set to 1 mL h^−1^, and the distance between the needle tip and the collector plate was fixed at 12 cm. The resulting fiber membranes were vacuum-dried at room temperature for 24 hours.

### 2.7 Characterization

The product structure was characterized using a Fourier Transform Infrared Spectrometer (TENSOR27). First, dried potassium bromide (KBr) powder was pressed into pellets. A small amount of the sample solution was then dropped onto the KBr pellet. The scan was performed over a frequency range of 550 to 4000 cm^−1^, with a resolution of 4 cm^−1^, 32 scans, and a wavenumber range from 4000 cm^−1^ to 400 cm^−1^. Additionally, attenuated total reflectance Fourier transform infrared spectroscopy (ATR-FTIR) mapping analysis of the product was conducted using a Nicolet™ iN10 microscope. The measurements were carried out with a resolution of 8 cm^−1^, a sampling interval of 3 seconds, and 16 scans per point, over a wavenumber range of 4000 cm^−1^ to 700 cm^−1^. The scanned area was 140 × 140 µm, with a step size of 10 µm, resulting in a 15 × 15 point mapping grid.

^1^H-NMR spectra were recorded on a Bruker AVANCE III spectrometer operating at 400 MHz, using TMS as the internal standard. Deuterated trifluoroacetic acid (TFA-d) was used as the solvent for PA4 and PEA samples, while deuterated chloroform (CDCl_3_) was used for PLA-SH samples. The acquired spectra were processed and analyzed using MestReNova software.

Samples were analyzed using a XploRA™ PLUS laser confocal micro-Raman spectrometer. Measurements were performed with a laser power set to 10%, an excitation wavelength of 785 nm, an exposure time of 3 seconds, and 10 accumulations per scan. The Raman shift range was from 3948 cm^−1^ to 200 cm^−1^.

The molar mass and dispersity of PLAL-SH were analyzed using a Bruker Autoflex Speed matrix-assisted laser desorption/ionization time-of-flight mass spectrometer (MALDI-TOF MS). Formic acid was used as the solvent, with a sample concentration of 5 mg mL^−1^. The matrix was 2,5-dihydroxybenzoic acid (10 mg mL^−1^) dissolved in tetrahydrofuran (THF). Measurements were conducted in linear positive ion reflector mode with an acceleration voltage of 20 kV and a vacuum pressure of 5 × 10^−7^ mbar.

The elemental composition of PEA, PLA-SH, and PA4 was analyzed using a PHI 5000 VersaProbe I X-ray photoelectron spectrometer (XPS). Both survey scans and high-resolution scans of the C 1s, N 1s, O 1s, and S 2p regions were performed for each sample.

Differential scanning calorimetry (DSC) measurements were conducted on a DSC8000 thermal analyzer under a nitrogen flow rate of 20 mL min^−1^. The samples were heated from 0 °C to 275 °C at a heating rate of 10 °C min^−1^.

X-ray diffraction (XRD) measurements of PEA and its electrospun fiber membranes were performed at room temperature using Cu Kα radiation with a wavelength of 0.154 nm. The X-ray tube was operated at 40 mA current and 40 kV voltage. The scanning range was from 5° to 35° (2*θ*) with a scanning speed of 5° per minute.

The microstructure of the electrospun fiber membranes was observed using a Hitachi SU8010 scanning electron microscope (SEM). Prior to imaging, the fiber membranes were gold-sputtered. An accelerating voltage of 30 kV was applied during observation. The average fiber diameter and its dispersity were estimated using ImagePro Plus 6.0 analysis software.

Mechanical properties of the electrospun fiber membranes were tested using a universal tensile testing machine (model CMT-4204). For each sample, five parallel specimens were prepared. The fiber membranes were cut into strips measuring 5 mm × 30 mm, and their thicknesses were measured. Both ends of the membrane strips were clamped in the machine's film grips for tensile testing, with a tensile speed of 5 mm min^−1^.

## 3. Results and discussion

**Fig. 1:**
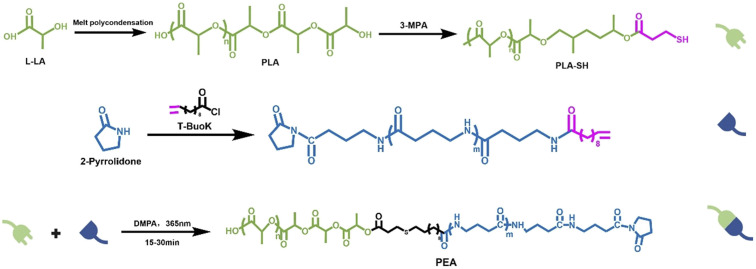
Thiol–ene click reaction for the synthesis of poly(ester amide) copolymers.

Using lactic acid and 3-mercaptopropionic acid as monomers, end-thiolated polylactic acid (PLA-SH) was synthesized *via* melt polycondensation; vinyl undecenoyl chloride was employed to initiate anionic ring-opening polymerization of butyrolactam to obtain vinyl-terminated PA4 with terminal double bonds; subsequently, polythiolated PLA and vinyl-terminated PA4 were copolymerized *via* thiol–ene click reaction to prepare PEA materials (Fig. 1).

### 3.1 Synthesis of PLA-SH

Thiol-functionalized polymers are important functional materials in the field of biomacromolecular engineering. The strategy of introducing thiol groups at the polymer chain ends holds significant research value in the construction of drug delivery systems and tissue engineering scaffolds. Therefore, developing a simple, safe, and efficient method for terminal thiol modification of polymers is of great importance.^34–36^ The melt polycondensation process does not require the use of toxic solvents or initiators, making it a green synthetic approach that offers clear advantages over ring-opening reactions in terms of operational simplicity, reaction safety, and product controllability. As shown in Fig. 1, the molar mass was regulated by adjusting the post-polycondensation time of polylactic acid (PLA) during melt polycondensation. Subsequently, thiol end-group modification of PLA was achieved through an esterification reaction between 3-mercaptopropionic acid and the terminal hydroxyl groups of PLA. The molar mass and dispersity of the resulting PLA-SH were determined by MALDI-TOF MS. Structural analysis was performed by examining the mass-to-charge (*m*/*z*) ratios. As shown in Fig. S1(a), the spectrum exhibits a consistent peak interval of 72 *m*/*z*, which corresponds exactly to the molar mass of a single lactic acid repeat unit, confirming that the PLA backbone consists of repeating lactic acid units.^37^ In addition, a signal interval of 88 *m*/*z* was observed, corresponding to the esterification between the carboxyl group of 3-MPA and the hydroxyl end group of PLA, indicating that 3-MPA was successfully incorporated to the PLA chain terminus.

**Fig. 2:**
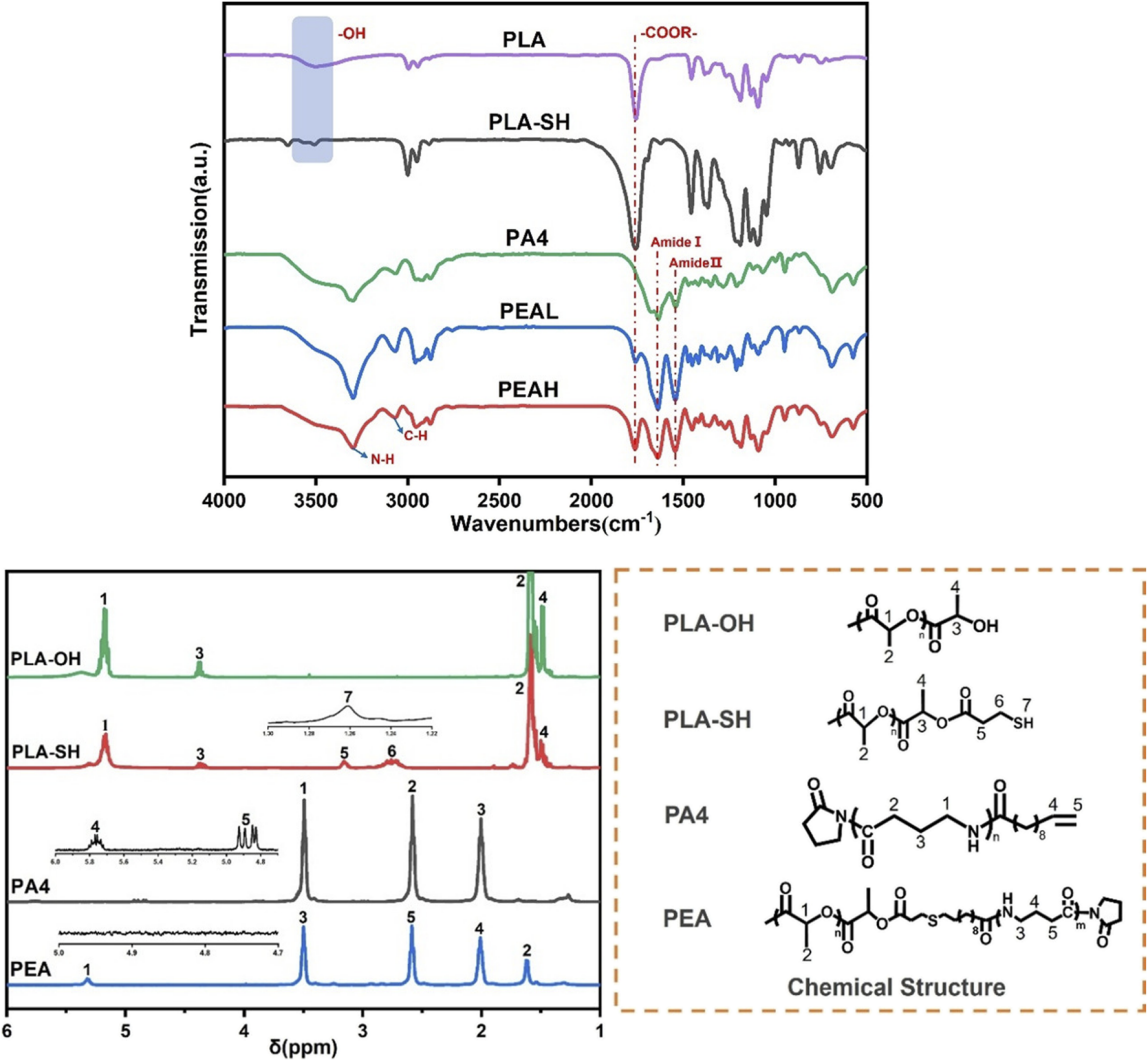
Structural characterization of PLA, PLA-SH, and PEA: (a) FTIR spectra, (b) ^1^H-NMR spectra.

Fig. 2(a) presents the FTIR spectra of all samples. For PLA-SH, the absorption bands observed at 3000–2900 cm^−1^ are attributed to the stretching vibrations of –C–H bonds, while the band at 1760 cm^−1^ corresponds to the stretching vibration of –C=O groups within the PLA backbone. The absorption band at 1382 cm^−1^ is assigned to the characteristic –CH_3_ group in PLA. Notably, compared with unmodified PLA, the –OH absorption band at 3497 cm^−1^ in PLA-SH is significantly weakened, indicating the consumption of terminal hydroxyl groups as a result of the esterification reaction. Since thiol groups exhibit weak characteristic bands in infrared spectroscopy and nonpolar functional groups typically produce stronger Raman scattering signals, Raman spectroscopy was employed to further confirm the presence of terminal thiol groups in PLA-SH. As shown in Fig. S1(b), a new Raman shift appears at 2580 cm^−1^ in the modified PLA-SH sample, which can be attributed to the stretching vibration of thiol groups.^38^ The typical ^1^H NMR spectra of PLA-OH and PLA-SH are shown in Fig. 2(b). In PLA-OH, the main peaks at 1.59 ppm and 5.17 ppm correspond to the methyl (2) and methine (1) protons of the PLA repeating units, while the signals at 4.38 ppm and 1.48 ppm arise from the methylene (3) and methyl (4) protons of PLA. After modification with the terminal thiol group, PLA-SH exhibits new proton signals at 3.16 ppm and 2.76 ppm, which are assigned to the methylene protons on the thiol group at positions (5) and (6), respectively. A signal at 1.26 ppm is attributed to the protons of the terminal thiol group (7), indicating the successful attachment of 3-mercaptopropionic acid to the PLA chain end. The number-average molar mass (Mn) and degree of polymerization of PLA-SH were estimated using eqn (1), which is based on the ratio of the integral areas of the proton signals at positions (5) and (1), denoted as S5 and S1, respectively. The results are summarized in Table 1.

1 MnPLA − SH = 144S1/S5 + 178

**Table 1:** Molar mass and related parameters of PLAL-SH, PLAH-SH, and PA4

Sample	Mn^a^		DP^a^		Molar ratio of PA4/PLA^b^
	PLA	PA4	PLA	PA4	
PEAL	971.1	2503.5	11	26	2.48
PEAH	1845	2503.5	23	26	1.11

a Determined and calculated from ^1^H-NMR spectra.

b Calculated by comparing the integral areas of the PLA and PA4 blocks in the PEA ^1^H NMR spectra.

### 3.2 Synthesis of terminal vinyl-functionalized PA4

The synthesis of terminal vinyl-functionalized PA4 was carried out following previously reported methods,^32^ Chen *et al.* pointed out that using 10-undecenoyl chloride as an initiator helps to avoid the electron-withdrawing effect caused by amide bonds, thereby ensuring the progress of the “click” reaction. Fig. 2(a) shows the FTIR spectrum of the terminal vinyl-functionalized PA4. The absorption band at 3293 cm^−1^ corresponds to the N–H stretching vibration of associated PA4, while the band at 3066 cm^−1^ is attributed to N–H bending vibration. The amide I and amide II bands appear at 1640 cm^−1^ and 1540 cm^−1^, respectively. The ^1^H-NMR spectrum of terminal vinyl-functionalized PA4 is presented in Fig. 2(b). The three main signals between 2.1 and 3.6 ppm correspond to the protons of the three repeating methylene groups along the PA4 backbone. The signals at 5.76 ppm and 4.89 ppm are assigned to the protons (4) and (5) of the terminal vinyl group (–CH=CH_2_), respectively. Both FTIR and NMR results indicate that 10-undecenoyl chloride successfully initiated the anionic ring-opening polymerization of pyrrolidone and was incorporated at the chain end. The Mn and degree of polymerization of PA4 were estimated using eqn (2), based on the ratio of the integral areas of the proton signals at positions (1) and (4), denoted as S1 and S4, respectively. The results are summarized in Table 1.

2 Mn(PA4) = 42.5S1/S4 + 251

### 3.3 Synthesis of block PEA

The thiol–ene click reaction is a highly efficient, regioselective reaction between thiol groups and carbon–carbon double bonds. It proceeds rapidly under UV irradiation without the need for metal catalysts, and offers several advantages including high selectivity and the absence of by-products. Due to these features, it holds great promise for applications in biomedical materials. Moreover, performing click reactions between distinct polymer blocks facilitates structural control and can influence the final properties of the resulting materials.^39,40^ As shown in Fig. 1, block-structured PEA copolymers were synthesized *via* a thiol–ene click reaction between terminal thiol-functionalized PLA and terminal vinyl-functionalized PA4. An excess amount of vinyl-terminated PA4 was added to increase the probability of collisions between reactive groups. The unreacted PA4 was removed using methanol containing 10% calcium chloride, a solvent suitable for PA4. Fig. 2(b) presents the ^1^H NMR spectrum of PEAL, in which the characteristic proton signals corresponding to the two block repeating units can be clearly identified. Compared with the alkenyl-terminated PA4, the disappearance of the (4) and (5) proton signals of –CH=CH_2_ at 5.76 ppm and 4.89 ppm in PEAL indicates the successful occurrence of the “click” reaction. The actual molar ratio of PLA to PA4 in the copolymer was determined by comparing the integral areas of the (1) and (3) proton signals in PEA. The molar ratio of PA4 to PLA repeating units in the copolymer was approximately 2.48, which is nearly consistent with the theoretical value derived from the degrees of polymerization of the individual blocks (DP_(PA4)_/DP_(PLA)_ = 26/11). This agreement indicates that the composition of the two segments in the obtained polymer corresponds well to the designed feed ratio, further confirming the successful synthesis of the target PLA-*b*-PA4 block copolymer. The molar mass and compositional data of all samples are summarized in Table 1.

The Raman spectrum in Fig. S1(b) further supports the formation of the block copolymer. The characteristic vibrational peak of terminal thiol groups in PLA-SH disappears after the reaction, and a new peak at 1760 cm^−1^ appears in the PEA spectrum, corresponding to the ester carbonyl stretching vibration. The FTIR spectrum of PEA, shown in Fig. 2(a), exhibits a characteristic ester carbonyl band at 1760 cm^−1^, attributed to PLA, as well as amide I and amide II bands at 1640 cm^−1^ and 1540 cm^−1^, respectively, corresponding to PA4. It is worth noting that in PEAH, the intensity of the ester carbonyl stretching band at 1720 cm^−1^ is stronger than that in PEAL, indicating an increased PLA block ratio in the copolymer. Fig. S2 shows the band fitting results of the N–H stretching vibrations in the 3500–3100 cm^−1^ region for PA4 and the block copolymers, aimed at investigating the effect of PLA chain length on intermolecular interactions within the copolymers. Detailed fitting data are summarized in Table S1. As the PLA chain length increased, the proportion of associated N–H in the copolymers decreased from 94.2% to 86.7%, and the corresponding band position exhibited a slight blue shift from 3297.2 cm^−1^ to 3298.3 cm^−1^. These results suggest that the incorporation of longer PLA segments disrupts the intermolecular hydrogen bonding between PA4 chains, leading to a reduced content of associated N–H groups. Zhou *et al.*^41^ investigated the hydrogen bonding interactions between *N*-methylacetamide and methyl acetate/ethyl formate. Molecular dynamics simulations demonstrated that the amide–amide hydrogen bonds between *N*-methylacetamide molecules exhibit greater stability than the hydrogen bonds formed between *N*-methylacetamide and ester carbonyl groups.

The XPS results further characterized the molecular structures of the samples. As shown in Fig. S3(a), the appearance of binding energy peaks corresponding to C 1s and O 1s in the full XPS spectrum of PLA, as well as C 1s, O 1s, and N 1s in that of PA4, confirms the successful synthesis of the polymers. In the full XPS spectra of the terminally modified product PLA-SH and the copolymers, in addition to the aforementioned elements, the high-resolution spectrum in the binding energy range of 156–174 eV (Fig. S3(b))clearly reveals the S2p peak associated with the thiol group, indicating the presence of sulfur. To further analyze the variation in chemical bonding with changes in PLA chain length, high-resolution O 1s spectra of PEAL and PEAH were deconvoluted (Fig.s S3(c) and (d)). The relative contents of C–O, C–O–C, and C=O bonds in the copolymers were examined. As the PLA chain length increased, the proportion of C–O and C–O–C bonds decreased from 36.8% to 31.6%. This decrease is attributed to the fixed chain length of the PA4 block and the reduced number of terminal –C–OH groups exposed as the PLA chain becomes longer, resulting in a lower C–O content. These XPS results collectively confirm the successful synthesis of all polymers and the incorporation of PLA segments with varying molar mass.

### 3.4 Thermal and crystallization properties

**Fig. 3:**
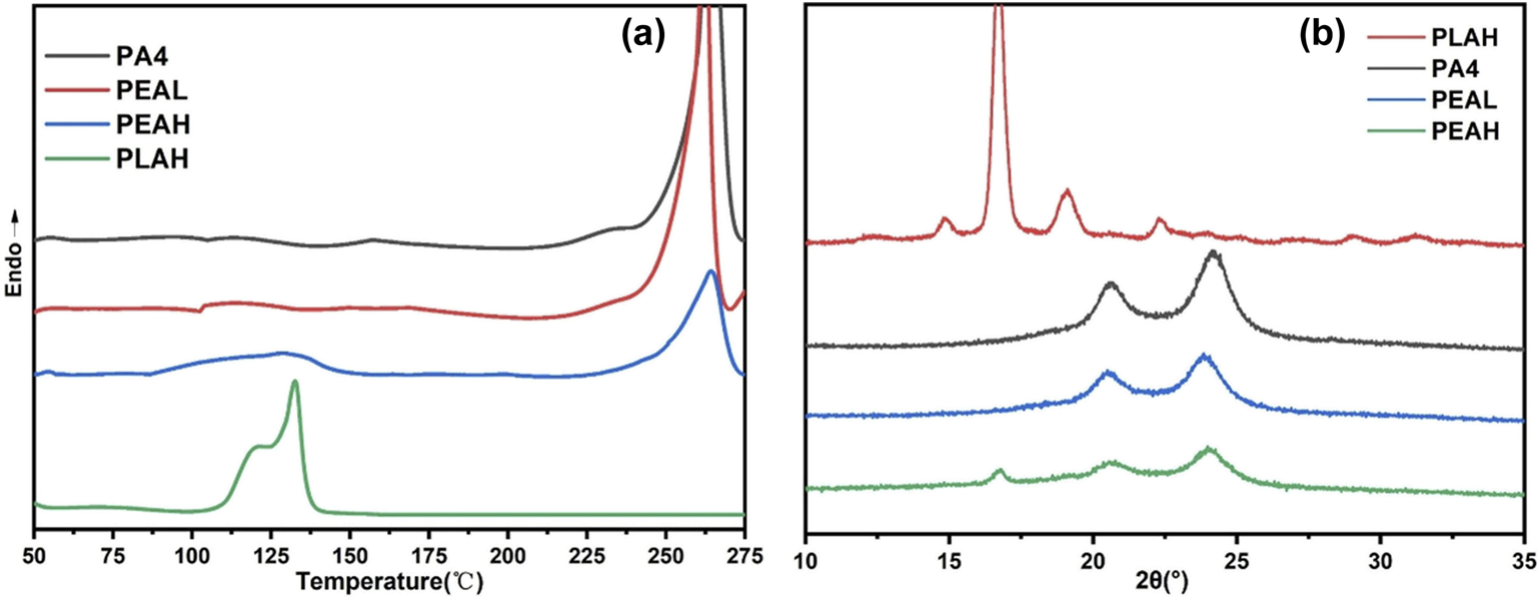
(a) First heating DSC curves of PLA, PA4, and PEA; (b) XRD patterns of PLA, PA4, and PEA.

Due to the close proximity between the decomposition temperature and the melting temperature of PA4, thermal degradation occurs during the melting process. Therefore, the first heating scan in differential scanning calorimetry (DSC) was used to investigate its melting behavior. Fig. 3(a) and Table 2 present the first heating curves and the corresponding thermal properties of all samples, respectively. As shown in Fig. 3(a), the glass transition of all samples is too weak to be clearly identified in the DSC curves. Both homopolymers exhibit distinct melting peaks: pure PA4 shows a melting temperature of 264.7 °C, while pure PLAH has a melting temperature of 132.7 °C. In contrast, no detectable melting peak is observed for PLAL, which is attributed to its low molar mass and the limited ability of its molecular chains to organize into a crystalline structure. For the copolymers, the crystallization and melting behaviors depend on their compositions. In PEAL, only a single melting peak at 262.3 °C, corresponding to the PA4 block, is observed, with a reduced enthalpy compared to pure PA4. As the PLA chain length increases, two distinct melting peaks appear in PEAL at 127.2 °C and 264.4 °C, which can be attributed to the PLA and PA4 blocks, respectively. The melting temperatures and enthalpies of both blocks are lower than those of their corresponding homopolymers. The DSC results suggest that insufficient PLA block length hinders its ability to crystallize effectively. Some of the short PLA chains may be incorporated into the crystalline domains of the polyamide segments as defects, leading to decreased melting temperature and enthalpies. With increasing PLA chain length, the PLA block begins to form its own crystalline domains. Due to the immiscibility between the PA4 and PLA blocks, the results suggest that phase separation may occur upon melting, allowing the two blocks to crystallize independently. However, their crystallization processes mutually interfere with each other, the crystallinity of the PLA block decreased significantly from 48.6% to 9.8%, indicating a strong inhibitory effect on the crystallization of both components within the copolymer.^42,43^

**Table 2:** DSC data of samples during the first heating scan

Sample	PLA			PA4	
	*T*_m_ (°C)	Δ*H*_m_ (J g^−1^)	*X*_c_ a (%)	*T*_m_ (°C)	Δ*H*_m_ (J g^−1^)
PA4	—		—	264.7	97.98
PLAH	132.7	45.5	48.6	—	
PEAL	—		—	262.3	88.1
PEAH	127.2	19.2	9.8	264.4	36.9

a The crystallinity estimated by comparing Δ*H*_m_ to the melting enthalpy of the perfect crystallization.

The crystalline structures of the samples were further analyzed by XRD, and the results are shown in Fig. 3(b). Characteristic diffraction peaks of PLA α and α′ crystal forms appear at 2*θ* = 14.8°, 16.6°, 19.1°, and 22.3°. For PA4, distinct diffraction peaks are observed at 2*θ* = 20.6° and 24.2°, corresponding to the (200) and (002) planes of the PA4 α-form crystal, respectively.^44,45^ Copolymers containing two or more blocks often form distinct crystalline structures; however, no new diffraction peaks were observed in the copolymers, indicating that no new crystal forms were generated. Only changes in the relative intensities of the existing diffraction peaks were detected. When the PLA block was short, no PLA crystalline peaks appeared in the XRD patterns of the copolymers, and the intensity of the PA4 crystalline peaks decreased, suggesting that the short PLA chains remained in an amorphous state and interfered with the crystallization of the PA4 segments. As the PLA block length increased beyond a certain threshold, diffraction peaks corresponding to PLA crystals began to appear. Nevertheless, the intensity of all crystalline peaks in the copolymers was lower than that of the corresponding homopolymers, indicating that the PA4 and PLA blocks crystallized independently within their own domains but mutually inhibited each other's crystallization. The crystallinity of the polylactic acid segments decreased from 48.6% to 50.5%. These results suggest that the two blocks retain their individual characteristics in the phase-separated crystalline domains. Previous studies have demonstrated that under similar conditions, the hydrogen bonding interactions between polyamide molecular chains result in a faster crystallization rate compared to polylactic acid.^46^ It can be inferred that, under the same conditions, the initially formed PA4 crystalline regions hinder the mobility of the PLA chains and restrict their crystallization, which is consistent with the DSC results. FTIR surface mapping further suggested the presence of phase separation behavior within the copolymers. As shown in Fig. S4, the spatial distribution of characteristic signals for PLA and PA4 segments implies a phase-separated morphology.

### 3.5 Electrospun fiber membranes of PEA

Solution electrospinning is a non-thermal processing technique used to fabricate continuous nanofibers, characterized by a high specific surface area and excellent porosity. It is widely applied in antibacterial food packaging, air filtration, and biomedical fields.^47,48^ Considering that PEA exhibits pronounced phase separation, its microphase structure is expected to confer advantages during the electrospinning process. Therefore, the synthesized PEA was applied to electrospinning to investigate its morphology and properties. It should be noted that the relatively low molar mass of PLA results in insufficient chain entanglement to reach the critical concentration required for electrospinning, thereby preventing the formation of continuous fibers.

The morphology of electrospun fibers is influenced by the concentration of the polymer solution. As shown in Fig. S5(a), the morphology of PA4 electrospun fibers varies with different concentrations. When the polymer solution concentration is 5 wt%, the pre-spinning solution exhibits low viscosity, and the concentration is insufficient to support the formation of stable electrospun fibers. As a result, the fibers are thinner, and part of the solution forms bead-like structures due to electrostatic spraying. With increasing solution concentration and viscosity, the bead structures disappear completely, the degree of molecular chain entanglement increases, and the fibers become thicker, continuous, uniform, and well-oriented (Fig. S5(b), (c)), with average diameters increasing to 97 ± 16 nm and 121 ± 31 nm, respectively. However, when the polymer solution concentration reaches 13 wt% (Fig. S5(d)), the fibers cannot be sufficiently stretched under the electric field. The high-viscosity polymer solution tends to resist elongation, leading to the formation of beads and an increased diameter distribution during fiber deposition.^49^

**Fig. 4:**
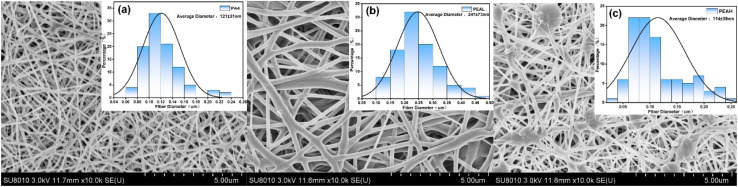
SEM images and diameter dispersity histograms of electrospun fibers from (a) PA4, (b) PEAL, and (c) PEAH.

SEM images and fiber diameter dispersities of electrospun membranes from different samples are shown in Fig. 4. Both PA4 and PEAL fibers exhibit smooth, slender, and bead-free morphologies, with their diameter dispersities following a unimodal pattern. In contrast, PEAH fibers display fiber bonding and bead formation. The average fiber diameters are approximately 121 ± 31 nm, 247 ± 73 nm, and 114 ± 39 nm for PA4, PEAL, and PEAH, respectively. Since the molar mass of the PA4 block in the copolymers is fixed, variations in fiber diameter are solely influenced by the length of the PLA segments. Compared to pure PA4 electrospun fibers, the diameter and diameter dispersity of PEAL fibers increase. This is attributed to the introduction of PLA blocks, which raises the overall molar mass of the copolymer and consequently increases the viscosity of the electrospinning solution, ultimately resulting in thicker electrospun fibers.^50^ Although a further increase in the molar mass of the PLA block can enhance chain entanglement in the spinning solution, changes in fiber diameter are also influenced by interchain interactions within the copolymer. As demonstrated in Section 3.1.3, increasing the PLA block molar mass weakens the hydrogen bonding between PA4 chains. This reduction in non-covalent intermolecular interactions among polymer chains leads to a decrease in the viscosity of the spinning solution, which in turn causes the formation of beads and a reduction in fiber diameter.^49,51,52^

The successful electrospinning of the copolymers, despite their relatively low molar mass, can be attributed to the structural characteristics of the PA4 segments, the high density of amide groups facilitates the formation of a robust intermolecular hydrogen-bonded network, which provides sufficient cohesive strength to maintain a stable jet. Furthermore, the amide groups are highly susceptible to polarization and protonation under a high-voltage electric field, resulting in an increased net charge density that enhances the drafting force required for fiber attenuation.

The XRD patterns of electrospun nanofibers from PEA and PA4 samples are shown in Fig. S6. Compared to the powder samples, the crystallinity of both PA4 and the PLA and PA4 blocks in the copolymers is significantly reduced in the electrospun fibers. Only the PA4 electrospun fibers exhibit weak diffraction peaks characteristic of the α-form crystal at 2*θ* = 20.4° and 24.1°. The PEA electrospun fibers remain nearly amorphous. In PEAL fibers with shorter PLA chains, a slight diffraction peak at 2*θ* = 20.3° can be observed. This phenomenon is attributed to the rapid solvent evaporation during electrospinning, which freezes molecular chain mobility. The solvent influx disrupts hydrogen bonding between PA4 chains, limiting the ordered stacking necessary for complete α-crystal formation. Moreover, hydrogen bonding interactions between PA4 segments and the solvent hexafluoroisopropanol (HFIP) further retard solvent evaporation. Consequently, PEA samples with higher PA4 content have sufficient time during electrostatic stretching to adjust molecular conformations and form crystals, whereas when the PLA block content is higher, PA4 chain alignment is restricted, and the fibers remain amorphous^32,48,53^

**Fig. 5:**
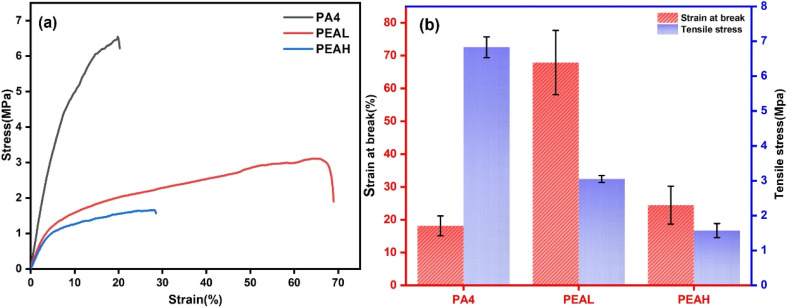
Mechanical properties of PA4, PEAL, and PEAH: (a) stress–strain curves; (b) tensile strength and elongation at break.

Fig. 5 presents the mechanical properties of the electrospun fiber membranes. Both PA4 and PEA fiber membranes exhibit similar mechanical behavior, showing linear elastic deformation up to the yield point. The tensile strength of pure PA4 fiber membranes reaches 6.83 MPa, with an elongation at break of 18.14%. This performance is attributed to the dense hydrogen bonding between molecular chains, which dissipates stress during stretching, allowing the membranes to maintain good strength and moderate toughness even at relatively low molar mass. After the introduction of PLA, the tensile strength of the PEA fiber membranes decreases, while the elongation at break increases significantly. The elongation at break of PEAL fiber membranes reaches 67.91%, indicating that the PLA blocks act as soft segments that weaken the intermolecular interactions among PA4 chains, facilitating polymer chain mobility and ultimately enhancing the toughness of the fiber membranes.^54,55^ It is noteworthy that a further increase in the PLA molar mass leads to a decline in the mechanical performance of the fiber membranes. This deterioration is attributed to the formation of beads in the electrospun fibers, which constitute structural defects unfavorable for membrane reinforcement (Fig. 4(c)).^56^

## 4. Conclusion

The thiol-terminated modification of PLA was successfully achieved *via* melt polycondensation, and biodegradable PLA-*b*-PA4 block copolymers were synthesized through thiol–ene click reaction with vinyl-terminated PA4. DSC and XRD analyses demonstrated the immiscibility of PLA and PA4 blocks, resulting in distinct crystalline domains with mutually inhibited crystallization between blocks. During electrospinning, the crystallinity of all samples decreased. By tuning the molar mass of the PLA block, a transition from brittle to tough fiber membranes was realized. Compared to single-component PA4 electrospun fibers (diameter: 121 ± 31 nm), copolymer fibers containing short-chain PLA blocks exhibited increased fiber diameters (247 ± 73 nm) due to higher molar mass, along with enhanced elongation at break (67.91%). However, introduction of longer-chain PLA blocks weakened interchain interactions, causing bead defects in the fiber structure and a decline in mechanical performance. The prepared fully biodegradable electrospun fibrous membranes exhibit promising potential for applications in biomedicine, packaging materials, and filtration.

## Author contributions

Fan Mo: writing – original draft, writing – reviewing & editing, data curation, formal analysis. Chen Tian: formal analysis, investigation. Rongrong Ji: formal analysis. Shixing Dong: data curation. Linwei Liang: methodology. Ling Zhou: writing – review editing. Hao Wu: writing – review editing. Xipo Zhao*: conceptualization, writing – reviewing & editing.

## Conflicts of interest

The authors declare no competing financial interest.

## Supplementary Material

RA-016-D6RA01092J-s001

RA-016-D6RA01092J-s002

RA-016-D6RA01092J-s003

RA-016-D6RA01092J-s004

RA-016-D6RA01092J-s005

RA-016-D6RA01092J-s006

RA-016-D6RA01092J-s007

RA-016-D6RA01092J-s008

RA-016-D6RA01092J-s009

RA-016-D6RA01092J-s010

RA-016-D6RA01092J-s011

RA-016-D6RA01092J-s012

RA-016-D6RA01092J-s013

RA-016-D6RA01092J-s014

RA-016-D6RA01092J-s015

RA-016-D6RA01092J-s016

## Data Availability

All data supporting this study are included in the article and its supplementary information (SI). Supplementary information: supporting data and table described in this article. See DOI: https://doi.org/10.1039/d6ra01092j.
