# Supplementary figures and images for: Preparation of biodegradable poly(lactic acid)-b-polyamide 4 block poly(ester amide) and its electrospun fibers
Source: RSC Adv. 2026 Apr 20;16(22):20381–90. doi: 10.1039/d6ra01092j (PMC13093889; doi:10.1039/d6ra01092j)

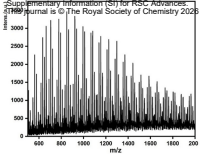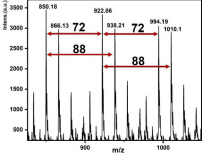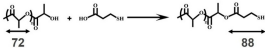

Supplement: RA-016-D6RA01092J-s001 [file RA-016-D6RA01092J-s001.pdf]

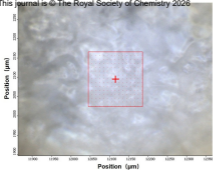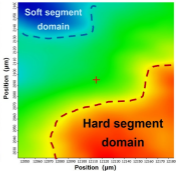

Supplement: RA-016-D6RA01092J-s002 [file RA-016-D6RA01092J-s002.pdf]

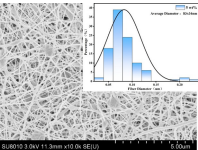

Supplement: RA-016-D6RA01092J-s003 [file RA-016-D6RA01092J-s003.pdf]

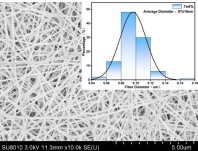

Supplement: RA-016-D6RA01092J-s004 [file RA-016-D6RA01092J-s004.pdf]

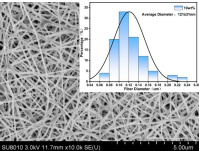

Supplement: RA-016-D6RA01092J-s005 [file RA-016-D6RA01092J-s005.pdf]

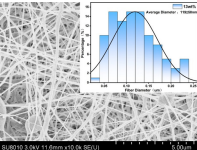

Supplement: RA-016-D6RA01092J-s006 [file RA-016-D6RA01092J-s006.pdf]

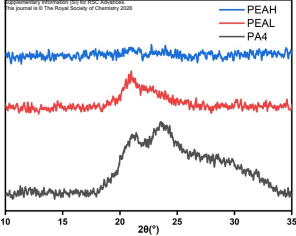

Supplement: RA-016-D6RA01092J-s007 [file RA-016-D6RA01092J-s007.pdf]

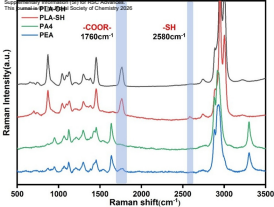

Supplement: RA-016-D6RA01092J-s009 [file RA-016-D6RA01092J-s009.pdf]

# PA4

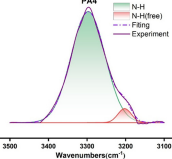

Supplement: RA-016-D6RA01092J-s010 [file RA-016-D6RA01092J-s010.pdf]

## PEAL

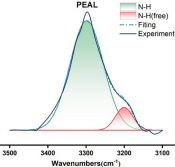

Supplement: RA-016-D6RA01092J-s011 [file RA-016-D6RA01092J-s011.pdf]

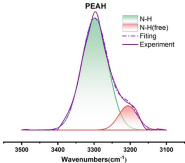

Supplement: RA-016-D6RA01092J-s012 [file RA-016-D6RA01092J-s012.pdf]

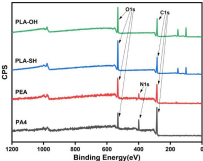

Supplement: RA-016-D6RA01092J-s013 [file RA-016-D6RA01092J-s013.pdf]

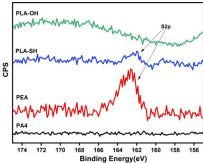

Supplement: RA-016-D6RA01092J-s014 [file RA-016-D6RA01092J-s014.pdf]

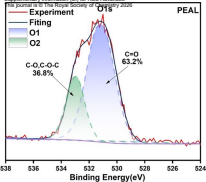

Supplement: RA-016-D6RA01092J-s015 [file RA-016-D6RA01092J-s015.pdf]

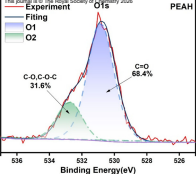

Supplement: RA-016-D6RA01092J-s016 [file RA-016-D6RA01092J-s016.pdf]
