# Supplementary material for: Preparation of biodegradable poly(lactic acid)-b-polyamide 4 block poly(ester amide) and its electrospun fibers
Source: RSC Adv. 2026 Apr 20;16(22):20381–90. doi: 10.1039/d6ra01092j (PMC13093889; doi:10.1039/d6ra01092j)
Supplement: RA-016-D6RA01092J-s008 [file RA-016-D6RA01092J-s008.pdf]

Supplementary Information (SI) for RSC Advances.
This journal is © The Royal Society of Chemistry 2026

Supporting Information

Preparation of Biodegradable Poly(lactic acid)-b-polyamide 4 Block Poly(ester amide) and Its Electrospun Fibers

Fan Mo[1,2], Chen Tian [1,2], Rongrong Ji [1,2], Shixing Dong [1,2], Linwei Liang [1,2], Ling Zhou [1,2,3], Hao Wu [1,2,3], Xipo Zhao [1,2,3,*]

(1. Hubei Provincial Key Laboratory of Green Materials for Light Industry, Hubei University of Technology, Wuhan, Hubei 430068, P.R.China; 2. New Materials and Green Manufacturing Talent Introduction and Innovation Demonstration Base, Hubei University of Technology, Wuhan, Hubei 430068, P.R.China; 3. Hubei Longzhong Laboratory, Xiangyang, Hubei 441000, P.R.China)

(Correspondence to: Xipo Zhao (E-mail: xpzhao123@163.com)

Correspondence author:

Xipo Zhao (e-mail: xpzhao123@163.com ,Tel.+8613871320654)

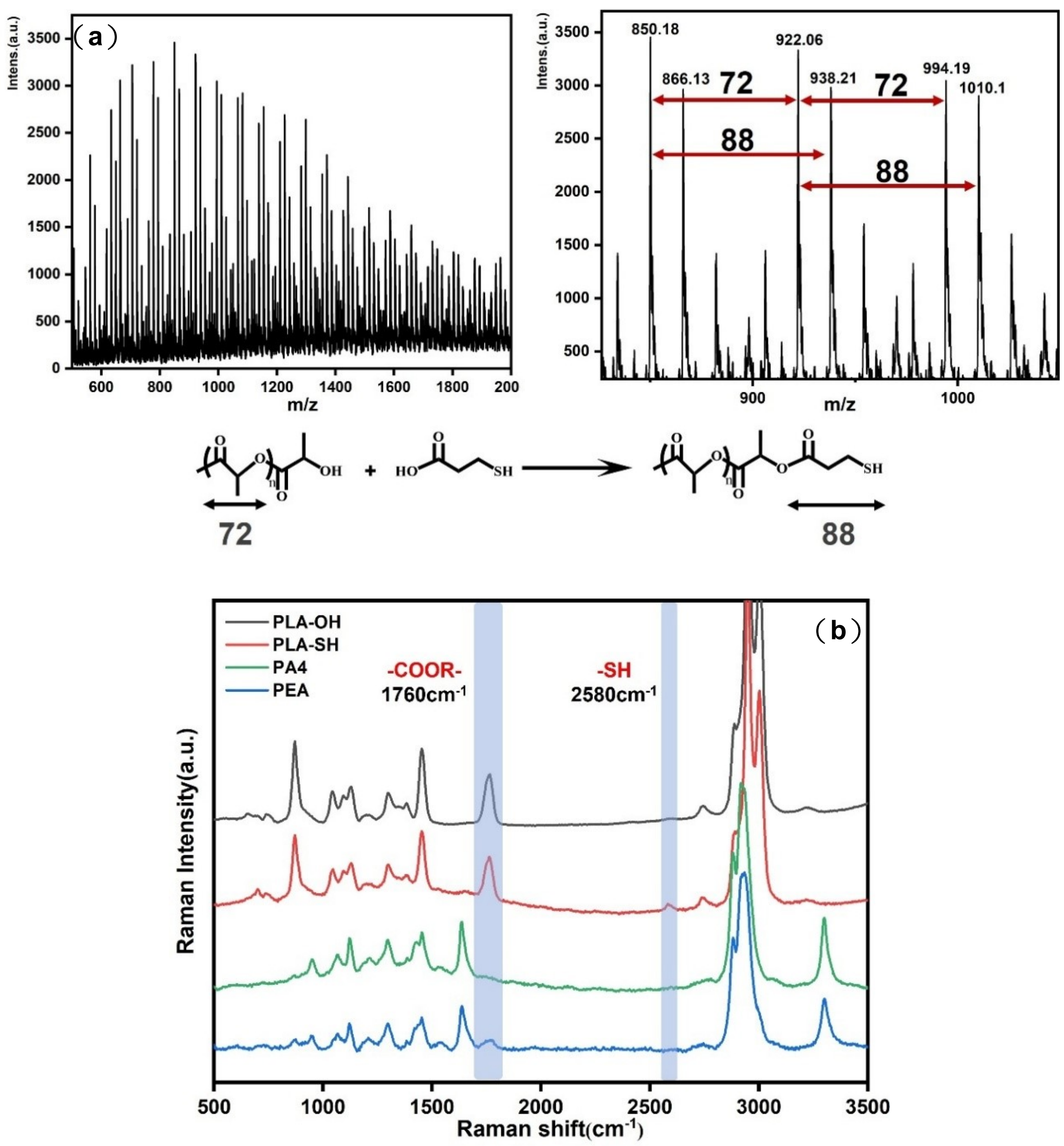

Figure S1 Structural characterization of PLA, PLA-SH, and PEA: (a) MALDI-TOF MS spectrum of PLAL-SH，(b) Raman spectra

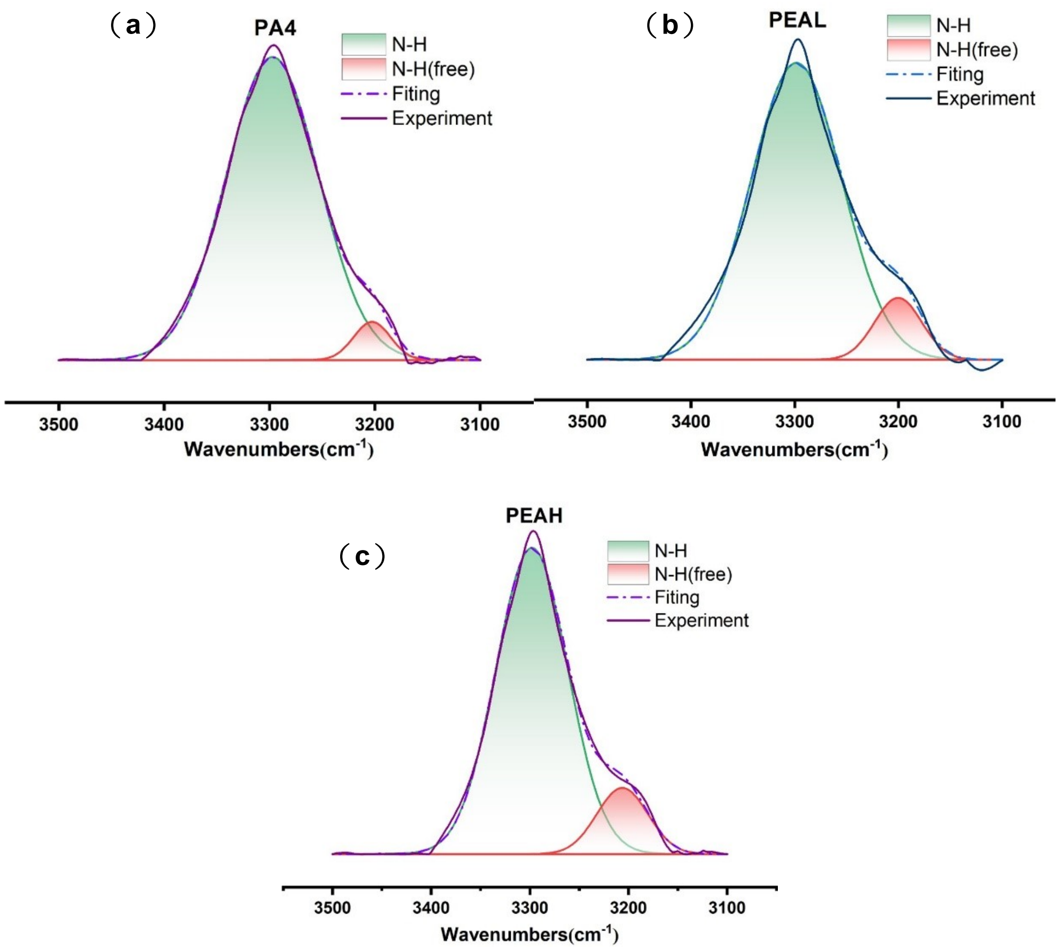

Figure S2 Band fitting results of infrared absorption in the 3500–3100 cm^{-1} region for (a) PA4, (b) PEAL, and (c) PEAH

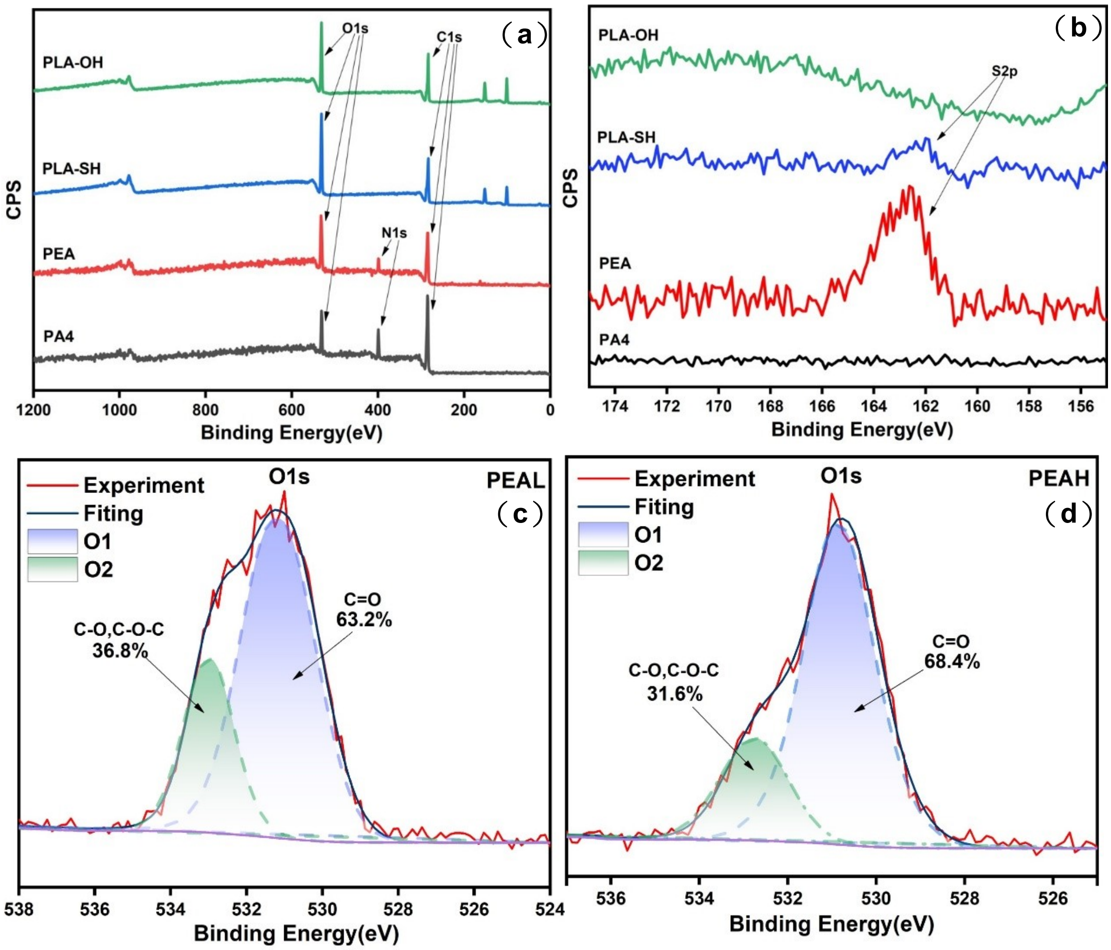

Figure S3 Wide-scan XPS spectra of the polymers; (b) Enlarged view of the wide-scan XPS spectra in the binding energy range of 156–174 eV; (c) High-resolution O1s XPS spectrum of PEAL; (d) High-resolution O1s XPS spectrum of PEAH.

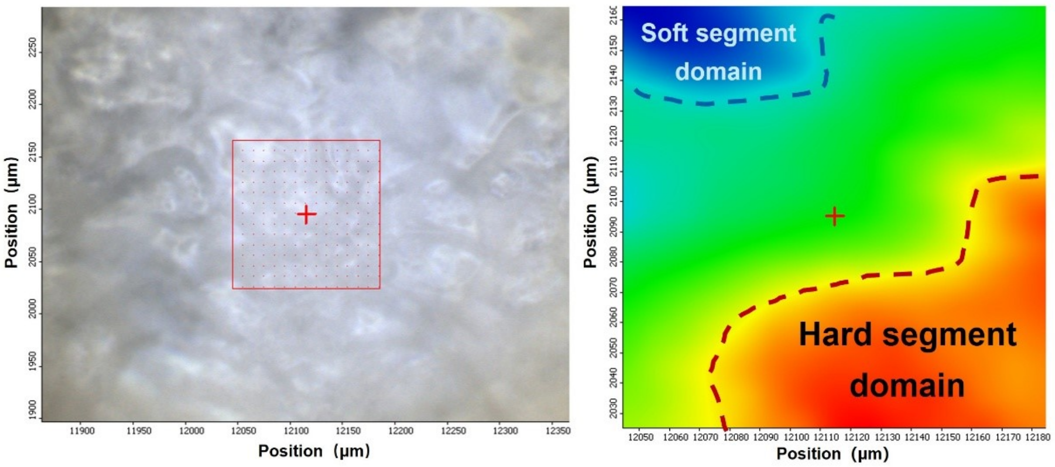

Figure S4 Optical image of the PEAH sample (left) and the 2D infrared surface mapping intensity image at 1640 cm^{-1} (right).

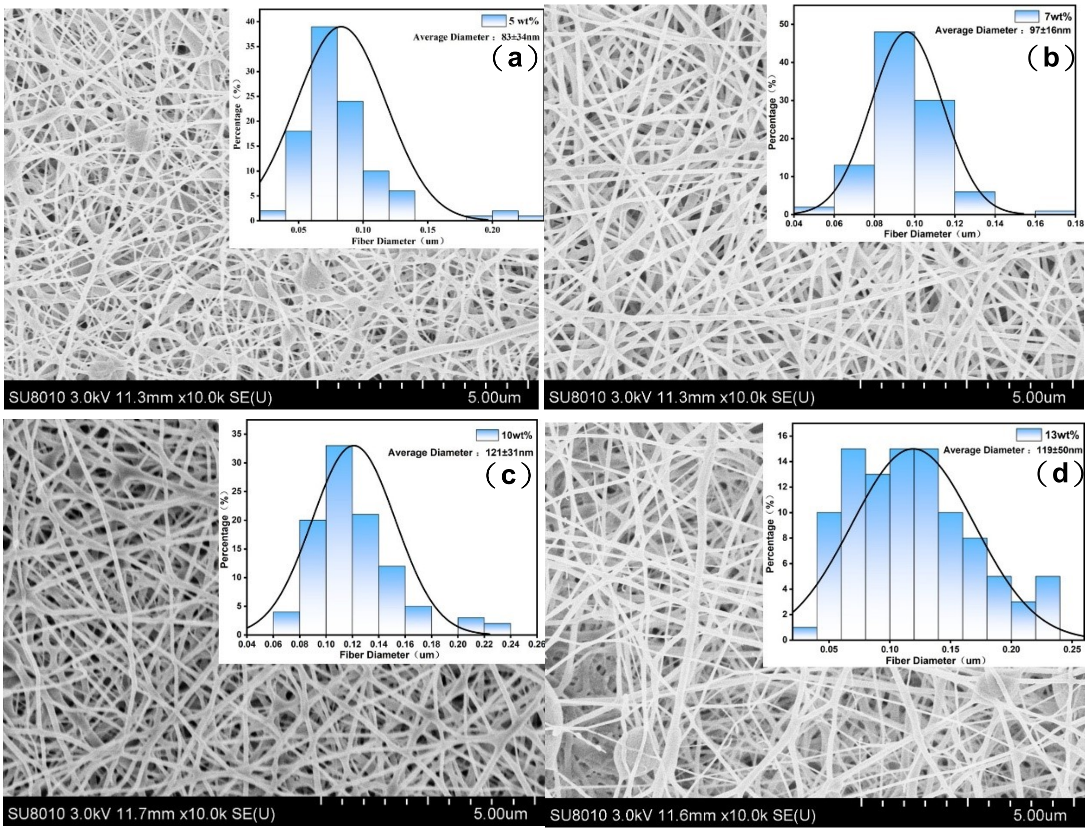

Figure S5 (a) Scanning electron microscope image of the morphology of PA4 electrospun fibers at a solution concentration of 5 wt %; (b) 7 wt %; (c) 10 wt % and (d) 13 wt %

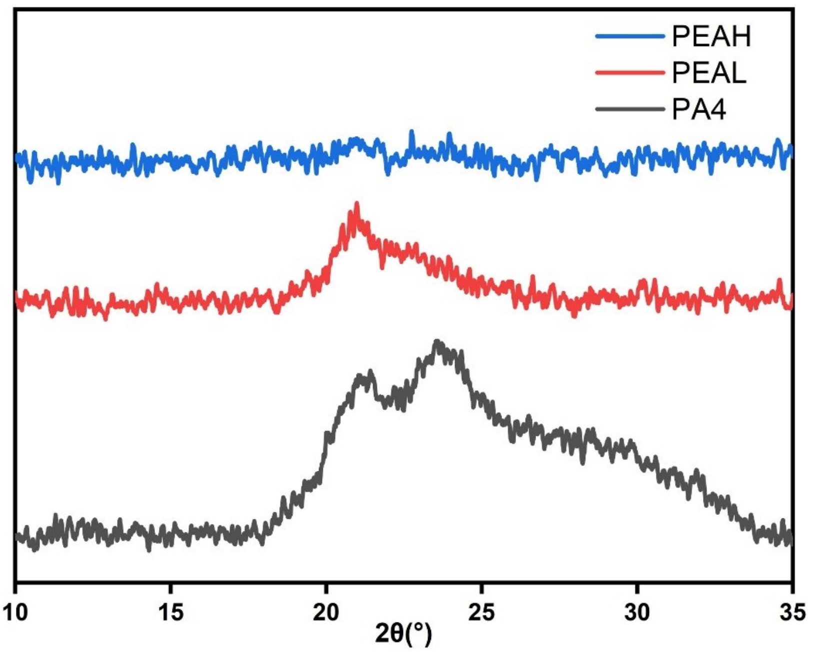

Figure S6 XRD patterns of electrospun fiber membranes of PA4, PEAL, and PEAH.

Table S1. Band fitting data of PA4, PEAL, and PEAH in the infrared absorption range of 3500–3100 cm^{-1}

**Sample**	**Type**	**Area%**	**σ/cm^{-1}**
PA4	N-H	94.2	3297.2
	N-H（free）	5.8	3202.5
PEAL	N-H	90.2	3298
	N-H（free）	9.8	3203.2
PEAH	N-H	86.7	3298.3
	N-H（free）	13.3	3206.2
